# The Role of AhR in the Hallmarks of Brain Aging: Friend and Foe

**DOI:** 10.3390/cells10102729

**Published:** 2021-10-13

**Authors:** Emmanuel S. Ojo, Shelley A. Tischkau

**Affiliations:** 1 Department of Pharmacology, Southern Illinois University, Springfield, IL 62901, USA; eojo74@siumed.edu; 2 Department of Medical Microbiology, Immunology and Cell Biology, Southern Illinois University, Springfield, IL 62901, USA

**Keywords:** aryl hydrocarbon receptor, AhR endogenous/exogenous ligands, brain aging hallmarks, neurodegenerative diseases

## Abstract

In recent years, aryl hydrocarbon receptor (AhR), a ligand-activated transcription factor, has been considered to be involved in aging phenotypes across several species. This receptor is a highly conserved biosensor that is activated by numerous exogenous and endogenous molecules, including microbiota metabolites, to mediate several physiological and toxicological functions. Brain aging hallmarks, which include glial cell activation and inflammation, increased oxidative stress, mitochondrial dysfunction, and cellular senescence, increase the vulnerability of humans to various neurodegenerative diseases. Interestingly, many studies have implicated AhR signaling pathways in the aging process and longevity across several species. This review provides an overview of the impact of AhR pathways on various aging hallmarks in the brain and the implications for AhR signaling as a mechanism in regulating aging-related diseases of the brain. We also explore how the nature of AhR ligands determines the outcomes of several signaling pathways in brain aging processes.

## 1. Introduction

Aging is an inevitable process in the human life cycle characterized by progressive deleterious changes in various anatomical and physiological functions [1]. These changes are the primary risk factors for various human diseases and death [2,3]. A rise in average life expectancy at birth in the US population from 78.54 years to 86.44 years by 2050 predicts an increased burden of age-related diseases [4], such as cancer, diabetes, cardiovascular diseases, and neurodegenerative diseases, which justifies a focus on aging research [5,6,7].

Similar to other organs, the brain also ages, which manifests as a decline in brain volume and cognitive function, as well as a decrease in motor coordination and decision-making [8,9,10]. Brain aging is hypothesized to be pivotal in the progression of neurodegenerative diseases and neuropsychiatric disorders that are prominent among older adults [11,12]. Several cellular and molecular pathways have been implicated in the progression of aging in various organisms, especially mammals [13]. These hallmarks of aging provide important clues that may serve as biomarkers and potential therapeutic targets to ameliorate the detrimental aspects of aging [14]. 

Recently, the aryl hydrocarbon receptor (AhR), an ancient protein that possesses highly conserved functions across various species, has been associated with aging and age-associated diseases [15]. Apart from its well-described role in xenobiotic metabolism, AhR signaling affects aging phenotypes and lifespan. For example, exposure to benzo(a)pyrene, an AhR ligand, promotes neurodegenerative disease-like syndromes in zebrafish [16]. Dietary factors, as well as microbiota by-products, that interact with AhR also modulate aging in *C. elegans* [17], indicating that AhR modulates aging in vertebrate and invertebrate species. Cellular development is also tightly linked with aging [18,19]. For instance, an increase in the number of cortical neurons during development helps increase the reserve of these cells during aging, thereby extending longevity [20]. Interestingly, the physiological functions of AhR include the regulation of cell growth and differentiation during development. This review focuses on the impact of AhR signaling events on various aging hallmarks in the brain, such as mitochondria toxicity, glial cell activation, inflammation, increased oxidative stress, etc. The activation of AhR pathways through various endogenous and exogenous ligands, and their influence on brain aging, is also explored. Finally, implications for AhR signaling as a component of age-related diseases of the brain, and its potential as a therapeutic target in neurodegenerative disease, are discussed.

## 2. AhR Expression, Functions, and Signaling in the Brain 

**Figure 1:**
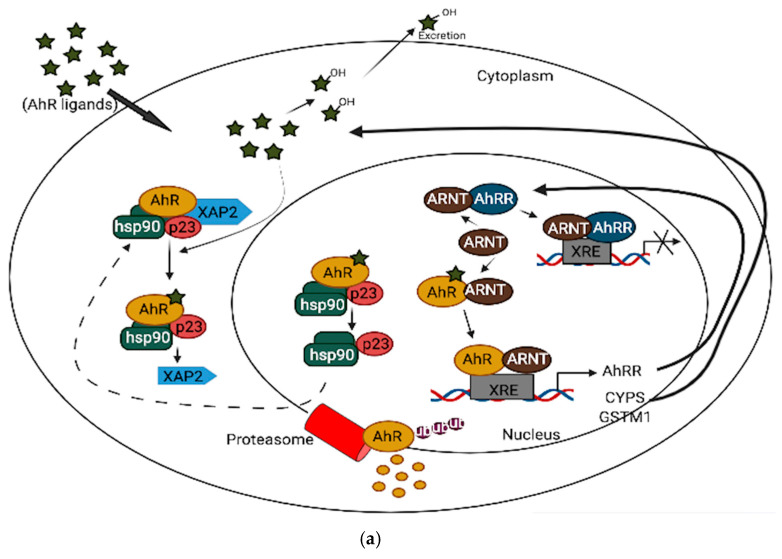

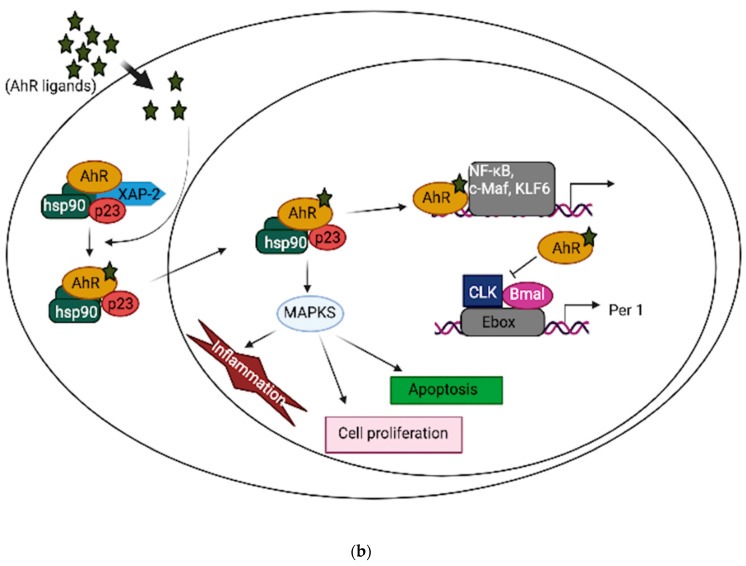
(**a**): AhR canonical pathway activation. (**b**): AhR non-canonical pathway activation.

AhR, a member of the basic helix-loop-helix (bHLH)-PAS superfamily, performs various functions within the brain [21]. It is an ancient protein that possesses shared functions and structures across various species in the evolutionary tree [22]. It is widely distributed in various regions of the brain, such as the hippocampus, the cortex, and the hypothalamus, and its expression changes during the course of brain development [23]. In neuronal progenitor cells, AhR interacts with its partners to direct differentiation into several neuronal subtypes, as well as to influence dendrite morphogenesis [24,25,26]. Although AhR expression decreases from the embryonic period into adult life [23,27], several physiological functions remain in the adult brain, which include the regulation of neurotransmitter levels, blood-brain barrier functions, and immune responses [28,29,30]. Furthermore, AhR contributes to glial cell and neuroendocrine system function [31,32]. AhR activation interacts at various levels in the neuroendocrine system, from the hypothalamus down to the target organ [31]. For example, the AhR agonist, 2,3,7,8-tetrachlorodibenzo-p-dioxin (TCDD) disrupts the secretion of several releasing hormones in the hypothalamus, such as corticotropin-releasing factor and vasopressin [33]. Furthermore, AhR activation in the brain leads to decreased estrogen receptors and estrogen levels [34,35]. Depending upon the ligand, AhR may act through different mechanisms to mediate its cellular and physiological functions [35]. AhR signaling is complex and broadly divided into canonical and non-canonical pathways. In the absence of ligands, AhR is predominantly found in a cytoplasmic complex with heat shock protein 90 (HSP90) dimers, HBV X-associated protein 2 (XAP-2), and p23 chaperone protein. However, in the canonical pathway, ligand activation of AhR leads to the dissociation of HBV X-associated protein 2 (XAP-2) from heat shock protein 90 (HSP90) in the cytoplasm; the activated AhR translocates into the nucleus, where it dimerizes with aryl hydrocarbon receptor nuclear translocator (ARNT) and binds to xenobiotic response elements (XREs) on the DNA, leading to the transcription of various cytochrome P450s (CYPs), and glutathione transferase (GST), which, among other events, feedback to metabolize the initial ligand. Some toxicological AhR ligands, such as TCDD and related compounds, are slowly metabolized following receptor induction, leading to persistent AhR activation [36]. Aryl hydrocarbon receptor repressor (AhRR), which is also an AhR target gene, helps mediate negative feedback through the sequestration of ARNT; ligand-activated AhR is subsequently degraded by the ubiquitin-proteasome system (Figure 1a). Apart from regulating phase 1 and phase 2 metabolic target genes for chemical defense, AhR also regulates several protein kinases, such as p21^Cip1^, and p27^kip1,^ that are necessary for organ development [37]. Inflammatory genes, such as Interleukin (IL)-6 and IL-1beta, and energy homeostasis genes, such as TCDD-inducible poly-ADP-ribose polymerase (TiPARP/PARP7), are also direct targets of AhR [37]. Thus, the target genes for AhR are broad, and many are unrelated to the toxicological functions of AhR. Physiologically, AhR may form alternative partnerships with other transcription factors, such as nuclear factor kappa-light-chain-enhancers of activated B cells (NF-κB), proto-oncogene c-Maf, Krueppel-like factor 6 (KLF6), and others, in the cytoplasm. For example, AhR interacts with NF-κB, which is involved in inflammation, immune and stress responses [38]; the induction of antioxidant genes requires the presence of both AhR and NF-E2 p45-related factor (Nrf2) at the promoter [39,40]. AhR also interacts with circadian clock components and intracellular signaling, such as the mitogen-activated protein kinase (MAPK) cascade involved in apoptosis, inflammation and cell senescence [41,42] (Figure 1b). ARNT shares similar sequences with brain and muscle Arnt-like protein-1 (BMAL1), a clock component, which may contribute to AhR/circadian clock interactions [43]. In HT22 hippocampal neuronal cells, the activation of AhR by α-naphthoflavone (α-NF) induces the phosphorylation of MAPK, leading to cell death in an AhR-dependent manner [44]. ARNT-2, a neuronal transcription factor that also belongs to the bHLH-PAS superfamily, is mainly expressed in the central nervous system and has been shown to be involved in neuronal survival [45]. Although ARNT-2s have been shown to form dimers with AhR in vitro [46], the question of whether ARNT-2 can interact with AhR in vivo remains, and is of importance to the understanding of whether ARNT-2 dimerization with AhR also participates in the activation of gene transcription in a similar way to AhR/ARNT in the brain and other organs.

Apart from xenobiotics, such as TCDD, and other polycyclic aromatic hydrocarbons (PAHs) that cross the blood-brain barrier (BBB) to mediate some of AhR’s effects in the brain, several endogenous tryptophan metabolites, such as kynurenine, serotonin, and 6-formylindolo [3,2-b] carbazole (FICZ), are implicated in AhR-related brain function and pathology [47,48]. Recently, attention has been drawn to the kynurenic pathway and microbial metabolites in the gut-brain axis, as well as central nervous system (CNS) development and diseases [48,49]. In the brain, L-tryptophan is primarily metabolized through kynurenic pathways, producing several ligands that bind to AhR [50]. AhR activation in glial cells by the microbial metabolism of dietary tryptophan interferes with the NF-κB inflammatory transcription program, thereby reducing neuroinflammation, which raises the possibility that this pathway could be targeted in neurodegenerative and autoimmune diseases in the CNS [51,52]. In addition to several gut microbiota metabolites, FICZ, an endogenous ligand of AhR, promotes neurogenesis in adult neurons, which is needed for hippocampal memory maintenance in mice. Several brain-related pathological conditions may also involve the non-canonical activation of AhR. For instance, in Alzheimer’s disease pathology, tryptophan derivatives (kynurenic acid and 5-hydroxyindole-acetic acid) can increase neprilysin expression, which is necessary for regulating amyloid beta clearance by proteolysis [53]. In neuronal cancers, such as glioma, AhR activation promotes a malignant phenotype by engaging transforming growth factor-β (TGF-β)/Smad [54,55]. Taken together, the available evidence suggests that AhR signaling plays a pivotal role in brain function and that its dysregulation may contribute to diseases of the brain. 

## 3. AhR and Aging Hallmarks in the Brain

### 3.1. Oxidative Stress

For years, the phenomenon of oxidative stress has been implicated in aging. Although several theories exist, the free radical theory of aging originally proposed by Denham Harman in the 1950s remains the most widely accepted, with modifications [56,57]. Aged tissues and senescent cells produce oxidative stress products, which lead to an imbalance between the oxidative and antioxidant defense network [58,59]. Besides, the exposure of cells to environmental oxidant generators, such as pesticides, heavy metals, and others, also contributes to this imbalance [60]. Just like other organs, a strong correlation exists between aging in the brain and increased reactive oxygen species (ROS) formation [61]; increased ROS can be attributed to mitochondrial dysfunction associated with aging [62,63]. Moreover, protein aggregation/modifications found in most aging-related brain diseases, including Alzheimer’s, have been attributed to increased ROS formation, which tends to impair proteasome and lysosome functions [64,65].

**Figure 2:**
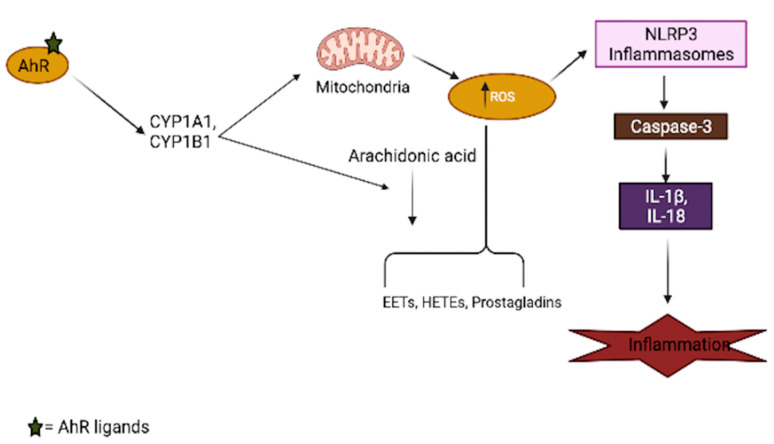
Involvement of AhR in oxidative stress generation. AhR activation by its ligands increases xenobiotic metabolism enzymes (CYPs), which results in mitochondrial toxicity, leading to the generation of reactive oxygen species (ROS). These enzymes also interact with the arachidonic acid pathway and increase the production of several arachidonic acid metabolites, such as EETs (epoxyeicosatrienoic acid), HETEs (hydroxyeicosatrienonic acid) and prostaglandins, which are sources of ROS in several tissues, including the brain. The generation of ROS in turn activates the inflammasome, which aids the secretion of inflammatory cytokines.

Aryl-hydrocarbon-receptor has been mechanistically shown to be involved in the generation of oxidative stress in the brain, as its activation by several ligands shifts the cellular redox balance towards favoring oxidative stress production [66,67,68]. The AhR agonist, TCDD, induces ROS production and oxidative DNA damage in astrocytes, leading to premature senescence, which is a hallmark of brain aging [69]. The generation of superoxide anions, the modulation of the CYP P450 system, mitochondrial dysfunction, and increased activation of arachidonic acid signaling are among the AhR-dependent pathways (Figure 2) that lead to increased ROS production in the brain [70,71]. Just like other organs in the body, the activation of AhR induces the expression of CYP1A1 and CYP1B1 in most brain regions, as well as the associated pituitary gland [72]; an increased expression of these xenobiotic metabolism enzymes can result in mitochondrial ROS production through an uncoupling process that leads to the release of superoxide and hydrogen peroxide (H_2_O_2_), which are believed to accelerate the aging process in the brain [73,74]. Increased production of ROS in mitochondria also regulates inflammasomes (NLRP3) by increasing the activation of inflammatory caspases in macrophages, which are necessary for cytokine synthesis, further contributing to brain inflammation [75]. In addition to the uncoupling process, arachidonic acid pathway activation by AhR leads to the increased generation of ROS through the metabolism of arachidonic acid by CYPs and other intracellular signaling processes [76,77]. 

Although AhR has also been implicated in antioxidant responses through its cross-regulation with Nrf2 in various tissues [39,40], the evidence for this pathway in the brain is yet to be fully established. The activation of AhR with the agonist, β-Naphthoflavone (BNF), has no significant effect on Nrf2 mRNA levels or antioxidant enzymes, such as glutathione transferase, in the brain regions of pigs [78]. In mice, the absence of AhR helps reduce oxidative stress in the brain [79]. Therefore, it is reasonable to suggest that the antioxidant role of AhR is either cell-specific and absent in the brain, or that the oxidant response overwhelms the antioxidant response in the brain.

### 3.2. Stress Response 

During stress, the body produces an adaptive response to reestablish the homeostasis that has been disrupted by the stressor [80]. Stress responses can either be cellular or generalized. The generalized stress response involves the release of glucocorticoids (stress hormone) via the neuroendocrine hypothalamic-pituitary axis. The cellular stress response involves various molecular changes, which may include the induction of heat shock proteins that are necessary for cell survival [81,82]. Brain aging can impose detrimental effects on both generalized and cellular stress responses, thus shifting away from an adaptive response towards a harmful effect. For instance, the age-related elevation of glucocorticoid levels contributes to hippocampal neuronal loss and cognitive impairment [82]. Postmortem cerebrospinal fluid in aged and Alzheimer’s patients contained elevated levels of cortisol [83], which suggests that the brain could be rejuvenated by inhibiting stress responses in the brain. Furthermore, organelle-specific stress response pathways and the ubiquitin proteasome system are also affected during aging [84]. Proteasome activities decline during aging, leading to increased protein modifications (a hallmark in various neurodegenerative diseases), which subsequently may reduce the effectiveness of the endoplasmic reticulum (ER) stress response [85]. Therefore, understanding stress response pathways during brain aging might provide relevant targets for therapeutic strategies in neurodegenerative diseases [86]. 

Aryl-hydrocarbon-receptor activation can modulate the neuroendocrine stress response system [31]. In the brain of rainbow trout, BNF acts through AhR signaling to downregulate steroidogenic acute regulatory protein, which is important for the biosynthesis of neurosteroids during stress. Furthermore, BNF suppressed pro-opiomelanocortin A (POMC-A), a precursor for adrenocorticotropic hormone (ACTH) that is necessary for the cortisol-induced stress response [87]. AhR also helps modulate the elevation in monoamine neurotransmitters that occurs during prolonged stress. For instance, AhR activation by PAHs and PAH-like compounds helps reduce cortisol and brain monoaminergic activities in rainbow trout after prolonged stress [88]. Cellular stress responses are also influenced by AhR activation [89,90], although these effects are yet to be explored specifically in the brain. Exploring AhR receptor involvement in glial cell cellular stress response mechanisms would be interesting, since these cells have been shown to be involved in brain stress responses [91,92].

### 3.3. Neurogenesis and Neuronal Plasticity

In the adult brain, neurogenesis appears to be important for the maintenance of the brain’s neuronal circuitry [89,93]. In the subgranular zone (SGZ) of the hippocampal dentate gyrus in young adult rats, newly generated neuronal cells tend to integrate with the pre-existing hippocampal circuit, which is necessary for learning and memory [94]. Neuronal stem/progenitor cells (NSC) are also found in the subependymal zones and olfactory bulbs of adult primates/humans [95,96]. Several neurodegenerative diseases, including Alzheimer’s disease, have been linked with aging-associated decline in neurogenesis and plasticity that occurs secondary to a loss in the proliferating potential of NSC [97,98]. Moreover, aged animals produce significantly fewer new neurons in the subventricular zone (SVZ) and SGZ of the hippocampus, which may contribute to a decline in cognitive functions that accompanies brain aging [99,100]. Aging also leads to the activation of glial cells and the subsequent secretion of pro-inflammatory cytokines, such as IL-1, which negatively impact NSC state and differentiation [100,101]. 

Aryl-hydrocarbon-receptor enhances neuronal proliferation during development; however, its role in adult neurogenesis is less well-investigated. AhR activation can regulate several genes involved in multiple aspects of synaptic plasticity and neurogenesis after brain development. A study using the Gene Ontology (GO) function and Kyoto Encyclopedia of Genes and Genomes (KEGG) enrichment analysis revealed that the administration of TCDD in the adult brain upregulates the genes required for synaptic plasticity and neuronal activities, including genes encoding for postsynaptic density 95 (PSD-95) protein and early growth response 1 (EGR1) [102]. The conditional deletion of AhR in adult mice also showed that AhR activation is necessary for SGZ neurogenesis by increasing the number of newborn granule cells in the DG of the hippocampus, which in turn improves hippocampus-dependent memory [103]. Similarly, AhR signaling helps restore neurogenesis after brain injury by enhancing ependymal glial cells to generate the new neurons necessary for repair in zebrafish [104]. Although several exogenous toxic AhR ligands have been studied for their neurotoxic effects targeting NSC in the adult brain, FICZ, an endogenous ligand of AhR, showed positive effects on the fate of NSCs by upregulating the ASCL1 and Ngn2 genes necessary for neuronal differentiation in the SGZ area of the adult mouse hippocampus [105]. Additionally, AhR activation by FICZ improves hippocampal-dependent memory and learning tasks, which [106] was reversed following treatment with the AhR antagonist, CH22319 [105]. Taking into account AhR activation and knockout studies, the normal physiological function of AhR in adult brain neurons is to enhance neurogenesis and synaptic plasticity. However, there is a possibility that the activation of AhR by toxic exogenous ligands might out-compete endogenous ligands for AhR binding due to their slow metabolism properties, and ultimately produce neurotoxic effects after the inappropriate or sustained activation of AhR.

### 3.4. Inflammation and Glial Cell Activation 

**Figure 3:**
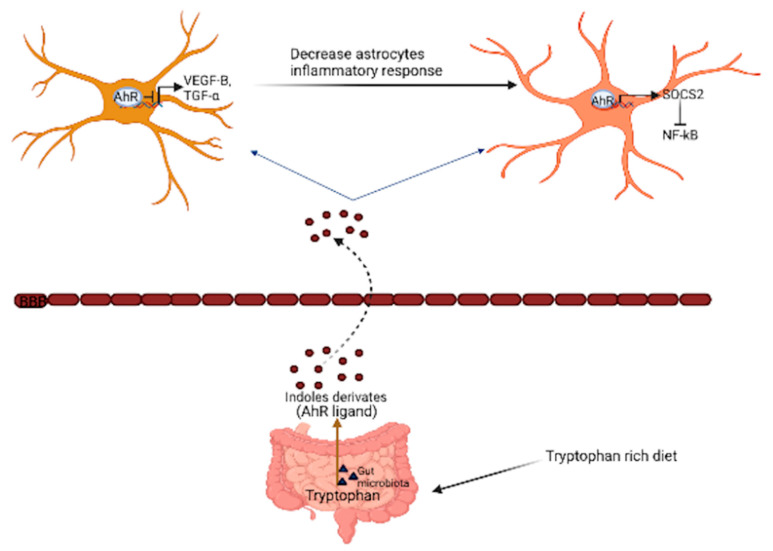
Suppression of CNS inflammation in glial cells through the activation of AhR by gut microbiota derivatives. Tryptophan metabolites, such as indole derivatives derived from the gut microbiota, influence CNS inflammation through the suppression of vascular endothelial growth factor B (VEGF-B) and TGF-alpha (transforming growth factor-alpha) in microglia cells. AhR activation by these metabolites also directly signals to SOCS2 protein (NF-κB inhibitor) in astrocytes.

Inflammaging, which is an excessive inflammation process that occurs during aging, results in several age-related diseases, such as Alzheimer’s disease, Parkinson’s disease, multiple sclerosis, cancer, and macular degeneration [107,108]. Inflammaging has also been linked with a depreciation in aged patients’ quality of life by increasing morbidity and mortality [109]. Aging in the brain is accompanied by increased pro-inflammatory cytokines, the downregulation of brain-derived neurotrophic factors (BDNF), and dysfunctional organelles that trigger a low-graded immune response, leading to changes in the morphology and functions of glial cells [110]. For example, aging alters the number of microglial cells in a region-specific manner [106]. In vivo and ex vivo approaches have shown that aged glial cells display a pro-inflammatory phenotype; RNAseq and early microarray analysis of astrocytes in aged mice indicate an increase in the neuroinflammatory A1-like reactive astrocyte phenotype when compared to young mice [111]. As with the astrocytes, transcriptional signatures for microglial activation are also present in the aged brain [112]. Thus, the collective evidence suggests that brain aging is accompanied by increased reactivity to glial cells, making the brain susceptible to neuroinflammation. During inflammation, NF-κB, which is responsible for pro-inflammatory cytokine transcription, binds directly to the promoter region of the AhR gene and induces its expression [113]. An in vitro study using lipopolysaccharide (LPS) to induce inflammation in glial cells shows increased AhR expression [32]. In addition, the tryptophan metabolite kynurenine, an AhR endogenous ligand, has been proposed as a biomarker for inflammation [114]. During aging, the blood kynurenine/tryptophan ratio becomes elevated, which is similar to observations of inflammatory-related disease states, including neurodegenerative diseases [115,116]. Native T cells that are involved in immune surveillance also express AhR, which, when activated by kynurenine, aids in the resolution of inflammation in several tissues by driving the differentiation of Tregs that secrete anti-inflammatory cytokines [117,118]. Dietary indoles, such indole-3-carbinol, and gut microbiota-derived indoles, such as indoxyl-3-sulfate, activate glial cells through AhR to mediate the response to CNS inflammation (Figure 3) [119,120]. These metabolites activate AhR, which in turn inhibits NF-κB by increasing the expression of SOCS2 protein (a suppressor of cytokine signaling) in astrocyte cells [121]. In microglia, AhR suppresses the NF-κB-driven expression of vascular endothelial growth factor B (VEGFB), reducing the activation of reactive astrocytes during inflammation. Consequently, targeting this pathway (AhR-NF-κB) might help reduce CNS inflammation [122,123]. However, the effect of exogenous AhR ligands on inflammation in the brain during aging has not been extensively studied. A recent study by Lowery et al. showed that TCDD exposure does not alter the morphology or inflammatory response of cortical microglia [124]. Nevertheless, more studies need to be performed to assess microglia activation in other regions of the brain following TCDD exposure, because the TCDD effects on glial cell activation might be region-specific. The long-term effects of AhR activation have not been studied. Moreover, a deficiency of AhR can also accelerate inflammaging. AhR-deficient mice exhibit several aged brain-related characteristics, such as enhanced hippocampal gliosis, increased plasma inflammatory cytokines, and accelerated hippocampal memory loss, at 16 months of age [125]. Clearly, the role of AhR in CNS inflammatory processes remains poorly understood.

## 4. AhR Signaling Mechanism in Aging-Related Brain Diseases

Compelling evidence indicates that AhR signaling pathways, especially after activation by endogenous AhR ligands (tryptophan metabolites), are involved in neurodegenerative diseases. Below is an overview of the effects of AhR signaling in prominent aging-related brain diseases.

### 4.1. Parkinson’s Disease (PD) 

PD is the second most common neurodegenerative disease, characterized by motor decline that occurs secondary to a loss of dopaminergic neurons [126]. The activation of AhR may provide protective effects in PD. The E3 ligase parkin directs the ubiquitination of proteins such as alpha-synuclein, Cdc- Rel, synphilin-1, and plays an important role in the progression of PD. Interestingly, parkin is an AhR target gene, induced by AhR activation in the ventral midbrain of mice, which in turn promotes the degradation of alpha-synuclein [127]. Toxic exogenous ligands such as TCDD increase the degeneration of dopaminergic neurons in the midbrain through increased oxidative stress, leading to PD. In contrast, several phytochemicals, such as tangertin, a citrus flavonoid, as well as natural compounds (Withanolide A, Withaferin A) from Withaferin Sominifera plants, act through AhR to protect against Parkinson’s symptoms in several models of PD [128,129]. An in vitro study also showed that the activation of AhR can induce tyrosine hydroxylase (TH) enzymes, which leads to increased dopamine and L-DOPA in murine neuroblastoma cells. In this same study, AhR was also detected in TH-positive neurons in the substantia nigra pars compacta (SNc), which are implicated in PD [130]. Given the evidence showing the neuroprotective effects of AhR against PD, further investigation should explore how several non-toxic AhR agonists might be used as a novel therapeutic strategy to delay or improve PD progression.

### 4.2. Alzheimer’s and Huntington’s

Alzheimer’s disease (AD), a neurodegenerative disease characterized by the aggregation of amyloid beta (Aβ) plaques that induce neuroinflammation and promote neuronal loss, has been linked with AhR. AhR levels in the post-mortem hippocampus and serum of AD patients are elevated when compared to those of young and elderly patients without dementia [131]. Furthermore, elevated levels of AhR and indoleamine 2,3-dioxygenase 1 (IDO1) enzymes are also present in the glial cells of post-mortem AD patient brains. Although AhR activation in glial cells is involved in the neuroinflammatory process [121], the relationship between glial AhR and neuroinflammation has not been explored. Duan et al. showed that aggregated Aβ activates AhR indirectly through increased IDO1, an enzyme responsible for the degradation of tryptophan, thereby accelerating the production of tryptophan metabolites that can act as AhR ligands to induce neurotoxicity via the Aβ-Kynurenine-AhR pathway [132]. Conversely, IDO1 enzyme inhibitors attenuate Aβ-AhR neurotoxic activities by reducing neuronal apoptosis and restoring the neuronal cytoskeleton. 

Both human and in vitro studies suggest that AhR might be involved in the AD neurodegenerative process. The contribution of environmental factors to AD risk has become an important aspect in AD research [133,134]. Because numerous environmental compounds may activate AhR, the evaluation of how these substances may interact with AhR as a mediator of AD risk alongside aging remains an interesting and open research question.

In Huntington’s disease (HD), the absence of AhR improves the behavioral and neurological phenotype. Quatzalli et al., showed that a lack of AhR in R6/1 mice, a recognized mouse model of HD, helps reduce neuroinflammation by impairing astrogliosis and the rate of motor co-ordination deterioration [135]. Kynurenine pathway metabolites, which may activate AhR, are also implicated in HD. In the striatum of HD patients, kynurenine acid (KYNA) levels are significantly reduced [136]. The ablation of kynurenine-3-monooxygenase (KMO), an enzyme responsible for increasing the levels of tryptophan neurotoxic metabolites, increases the level of KYNA in several regions of the brain and peripheral organs of R6/2 HD mouse models [137]. Furthermore, knocking out KMO in this HD mouse model decreases the plasma levels of pro-inflammatory cytokines. However, although the levels of KYNA in animal models of HD remain unknown, they require investigation, since AhR-deficient mice demonstrated elevated KYNA levels and reduced responsiveness to quinolinic acid in a biochemical model of HD [74,79]. Understanding whether there is a link between AhR and KYNA levels might help to offer a better mechanistic explanation for the detrimental effects of AhR found in the R6/1 transgenic model of HD. Furthermore, exploring the effects of several AhR antagonists in the improvement of HD symptoms in several models would be of therapeutic value.

### 4.3. Multiple Sclerosis and Amyotrophic Lateral Sclerosis

AhR may be a therapeutic target in the treatment of multiple sclerosis (MS), a CNS autoimmune disease. Endogenous AhR agonists are reduced in serum derived from MS patients [123]. Although its mechanisms remain unknown, the altered gut microbiome in human MS provides an interesting avenue for investigation [138]. The single-nucleotide polymorphisms (SNP) of several AhR pathway genes are linked with MS, including the primary AhR target gene, CYP1A1 which has been associated with the secondary progression of MS in genotyping analyses [139]. Moreover, AhR may limit CNS inflammation, a hallmark of MS, by negatively regulating astrocyte activation [140]. In autoimmune encephalomyelitis (EAE), an animal model of MS, treatment with laquinimod reduced astrogliosis and prevented downstream pro-inflammatory cytokines in an AhR-dependent manner [141]. Environmental factors also contribute to decreased AhR protective activities in MS pathophysiology. For example, a risk factor for developing MS, smoking, leads to increased demethylation of aryl hydrocarbon receptor repressor, leading to the inhibition of AhR signaling pathways and subsequent increases in CNS inflammatory and neurodegenerative processes in MS [142,143].

In amyotrophic lateral sclerosis (ALS), TAR DNA binding protein 43 (TDP-43) aggregation occurs in the brain; drugs that target this protein have become a therapeutic approach to this disease [144]. The activation of AhR by either an exogenous (TCDD) or endogenous ligand (FICZ) increased the level of TDP-43 protein in human neuronal cell lines (BE-M17) and motor neurons differentiated from iPSCs; however, the observed effects were reversed by AhR antagonists, suggesting that exposure to environmental toxins that activate AhR can be a risk factor for ALS development/progression [145]. Although it is too early to make any conclusions about the detrimental effects of AhR activation in ALS, more studies using several ALS animal environmental and genetic models should be explored. 

## 5. Concluding Remarks 

The aggregated evidence demonstrates that the activation of AhR could be either beneficial or detrimental in brain aging; the effects depend on context, especially the type of ligand binding. Furthermore, changes in AhR activities during aging might contribute to the acceleration of brain aging processes. Exogenous, toxic AhR ligands could accelerate brain aging, while endogenous AhR ligands, especially those produced by the gut microbiota, may actually decrease the rate of aging. The activation of AhR by endogenous ligands may act as a homeostatic sensor through several physiological process that favor neuroprotective and glial cell physiological functions, rather than pathophysiological events associated with brain aging. Therefore, exploring the clinical relevance of compounds that activate physiological AhR signaling may provide new therapeutic targets in neurodegenerative disease. Furthermore, the investigation of AhR as a part of other age-related dysfunctions, including altered energy metabolism in the brain, and impaired proteasome and lysosome functions, among others, are clearly warranted in order gain a better understanding of how AhR may contribute to the pathophysiology of aging.
